# Percutaneous tracheostomy in patients with severe liver disease and a high incidence of refractory coagulopathy: a prospective trial

**DOI:** 10.1186/cc6143

**Published:** 2007-10-08

**Authors:** Georg Auzinger, Gerry P O'Callaghan, William Bernal, Elizabeth Sizer, Julia A Wendon

**Affiliations:** 1 Institute of Liver Studies, Liver Intensive Care Unit, King's College Hospital, Denmark Hill, London SE5 9RS, UK

## Abstract

**Introduction:**

The purpose of this study was to assess the safety of percutaneous dilational tracheostomy (PDT) performed by experienced operators in critically ill patients with liver disease and coagulopathy.

**Methods:**

We conducted a prospective cohort study in a 10-bed specialist liver intensive care unit of a tertiary university teaching hospital. The study consisted of 60 consecutive patients in need of tracheostomy insertion. Patients were categorized as having refractory coagulopathy if their platelet count was less than or equal to 50 × 10^9 ^cells/L or their international normalized ratio (INR) was greater than 1.5 on the day of PDT and for the 72 hours afterward despite clotting support.

**Results:**

Twenty-five patients fulfilled the definition criteria of refractory coagulopathy. There was no significant difference in the number of adverse incidents between groups. Only one patient in the coagulopathy group had a severe bleeding complication, but this did not require open surgical intervention. The rate of clinically relevant early complications in all patients was not higher than expected (*n *= 7, 12%). Resource utilisation was higher for patients with coagulopathy who received significantly more platelet transfusions over the 3-day period (80 versus 49 units; *p *= 0.009) and who demonstrated a trend toward increased fresh frozen plasma requirements (*p *= 0.059). The number of patients requiring platelet transfusion was higher in the coagulopathy group (21/25 versus 20/35; *p *= 0.029). Hospital survival did not differ between groups.

**Conclusion:**

PDT is safe and not contraindicated in patients with severe liver disease and refractory coagulopathy.

## Introduction

Since the introduction of guidewire-assisted percutaneous dilational tracheostomy (PDT) into routine clinical practice some 20 years ago by Ciaglia and colleagues [1], the technique or modifications thereof have been used increasingly in intensive care units (ICUs) worldwide. In fact, the procedure has replaced surgical tracheostomy in many ICUs given the ease and speed of application, lack of need for transfer to the operating theatre, and a comparable (if not better) safety profile [2]. With increasing familiarity with the procedure, indications for PDT have been extended to include patients with previously defined contraindications, such as unfavourable anatomy due to obesity or short neck [3], inability to extend the neck, and coagulopathy or use of anticoagulants [3,4].

Refractory coagulopathy and thrombocytopenia or impaired coagulation is frequently seen in patients with liver disease requiring ICU admission. A comprehensive, prospective risk assessment of PDT in this patient population has not been performed thus far. We report the results of a prospective study on the safety of PDT in patients with a wide range of liver disease, or following liver transplantation for acute liver failure, in which the incidence of refractory coagulopathy is high.

## Materials and methods

### Patients

Over a consecutive 7-month period, all patients requiring PDT in a 10-bed specialist liver ICU were enrolled in the study. The indication for tracheostomy was made by the consultant in charge of the ICU at the time. The procedure was carried out within 24 hours after the decision to perform PDT was made, regardless of the degree of coagulopathy and with clotting support as clinically indicated. No surgical tracheostomies were performed during the study period and no patients were excluded. Tracheostomies were performed at the bedside by experienced operators (at least 75 PDTs performed). The procedure was undertaken by one of two consultants and/or a single senior ICU trainee under consultant supervision. Informed consent from the patient's next of kin was obtained to undertake the percutaneous tracheostomy, and subsequently consent for the study was obtained. The study was approved by the local research ethics committee.

Bronchoscopic guidance was not routinely used. All patients received anaesthesia with propofol, midazolam, or lorazepam. Analgesia with fentanyl was administered to all but four patients. Atracurium or vecuronium was used for muscle relaxation. FiO_2 _(fraction of inspired oxygen) was increased to 1.0 and ventilator settings were kept unchanged, apart from patients on pressure support ventilation in which a controlled mode of ventilation, flow or pressure-limited, was initiated for the period of the intervention and maintained until reversal of paralysis. Continuous heart rate monitoring, arterial oxygen saturation measurement, end tidal CO_2_, and invasive arterial blood pressure monitoring were performed in all patients. Resuscitation and difficult airway equipment was present at the bedside.

Oxygenation and cardiovascular status, including use of inotropic or vasopressor support, was recorded prior to, during, and for a 48-hour period following the procedure. Twenty-four patients had advanced haemodynamic monitoring in place (transpulmonary thermodilution and pulse contour cardiac output monitoring via the PiCCO system; PULSION Medical Systems AG, Munich, Germany). In the latter group, volumetric preload markers (intrathoracic blood volume index, or ITBVI) and extravascular lung water index (EVLWI) were calculated.

Sixty patients underwent PDT during the study period. Forty-threetracheostomies were performed using the 'Blue Rhino' single-dilator technique (Cook Medical Inc., Bloomington, IN, USA). In the remaining 18 patients, the sequential dilation technique was used (Ciaglia Percutaneous Tracheostomy Introducer Set; Cook Medical Inc.). The procedure was performed as previously described [5,6].

### Definition of refractory coagulopathy and complications

Refractory coagulopathy was defined as a platelet count of less than 50 × 10^9 ^cells/L or an international normalized ratio (INR) of greater than or equal to 1.5 or a combination of both on the day of PDT and over the consecutive 3 days following PDT despite platelet transfusion and fresh frozen plasma (FFP) support. Patients were then defined as group 1 (refractory coagulopathy) or group 2 (mild or no coagulopathy).

The amount of platelet transfusions and FFP administered immediately before and during the 72-hour period following PDT was recorded. FFP and platelets were transfused in an attempt to raise the platelet count to greater than 70 × 10^9 ^cells/L and lower the INR to less than 1.5 prior to surgery. Standard doses of FFP (12 to 15 mL/kg) and platelets (1 unit of donor pooled platelets expected to raise the platelet count by 20 to 30 × 10^9 ^cells/L) were administered. INR and platelet thresholds were less stringent in the 72-hour period following PDT and were corrected as clinically appropriate.

Complications were defined as potentially life-threatening, severe, or minor and were further classified into early or late. Early complications referred to all immediate procedure-related adverse incidents occurring during PDT or in the subsequent 12 hours. Procedure-related death, cardiac arrest, posterior tracheal wall laceration, tension pneumothorax, loss of airway, and severe bleeding necessitating emergency transfusional support or open surgical intervention were classified as life-threatening. Hypotension requiring vasopressor support, significant hypoxaemia (sustained PaO_2 _[arterial partial pressure of oxygen] of less than 8 kPa), and false route were classified as severe complications. Minor complications not requiring any intervention included brief desaturation of less than 92%, temporary arterial hypotension (mean arterial pressure [MAP] of less than 60 mm Hg), lobar or segmental collapse not causing any respiratory compromise, mild local bleeding, and localized subcutaneous emphysema without evidence of pneumothorax or pneumomediastinum.

Life-threatening late complications included tracheal innominate artery fistula and tracheostomy cannula obstruction. Tracheostomy-related sepsis (stoma infection as the only identifiable cause for septicaemia), tracheomalacia, or clinically relevant tracheal stenosis (stridor following decannulation with confirmation of stenosis on computed tomography or following bronchoscopy) were classified as severe, and local stoma infection was considered a mild late complication. Transfusion of red packed cells in which another source of bleeding was evident, and in the absence of any stomal or intratracheal haemorrhage, was not deemed to be a complication of the procedure.

### Statistical analysis

Data are presented as median and range or as number and percentage as appropriate. Fisher exact and Mann-Whitney *U *tests were used to compare differences between patients with and without refractory coagulopathy.

## Results

### Patient demographics

Sixty patients underwent PDT over the course of a 7-month period. Patients in the coagulopathy group were older and had a higher Sequential Organ Failure Assessment (SOFA) score aetiology of underlying liver disease or duration of mechanical ventilation prior to PDT did not differ between groups (Table 1). Acetaminophen overdose was the most frequent cause for acute liver failure (*n *= 11), and there was a preponderance of alcoholic liver disease in the patients with chronic liver failure (*n *= 13).

**Table 1:** Baseline characteristics

	All	Coagulopathy (*n *= 25)	Mild or no coagulopathy (*n *= 35)	*P *value
Age in years	42 (16–80)	42 (16–80)	34 (16–63)	0.002^a^
Aetiology				
Acute liver failure	25 (42%)	10 (40%)	15 (43%)	
Chronic liver disease	19 (32%)	9 (36%)	10 (29%)	
Post-transplant	9 (15%)	5 (20%)	4 (11%)	
Other	7 (12%)	1 (4%)	6 (17%)	
Severity of disease				
APACHE II score	19 (4–30)	20 (9–28)	19 (4–30)	
SOFA score	13 (3–22)	14 (10–22)	12 (3–18)	0.002^a^
Duration of mechanical ventilation				
Duration of CMV^b^	7 (0–22)	7 (0–19)	6 (1–22)	
Tracheostomy method				
Blue Rhino^®^	43	17	26	

Values are presented as median (and range or percentage). ^a ^p < 0.05 significant. ^b^Duration of controlled mechanical ventilation in days prior to procedure. APACHE II, Acute Physiology and Chronic Health Evaluation II; SOFA, Sepsis-related Organ Failure Assessment.

### Incidence of refractory coagulopathy

Twenty-five patients fulfilled the definition criteria for refractory coagulopathy (group 1). All other patients had mild or no coagulopathy (group 2). Seventeen patients in group 2 had an INR of greater than or equal to 1.5 or a platelet count of less than or equal to 50 × 10^9 ^cells/L at least at one time point either on the day of or during the 72-hour period following tracheostomy. Only two patients had no coagulopathy, an INR of less than or equal to 1.2, and a platelet count of greater than 150 × 10^9 ^during the entire study period. Thirteen patients in group 1 had a platelet count of less than or equal to 30 × 10^9^.

### Patient outcome and complications

All but one patient survived the 72-hour observation period following tracheostomy. This was an elderly patient with cryptogenic cirrhosis and multiple organ failure in whom a decision was made not to escalate therapy. Multiple organ failure was unrelated to PDT and the patient died 72 hours after tracheostomy insertion. Table 2 shows the cumulative incidence of bleeding complications; this was not different between groups.

**Table 2:** Bleeding complications

	All	Coagulopathy (*n *= 25)	Mild or no coagulopathy (*n *= 35)	*P *value
Severe bleeding	1	1	0	NS
Local bleeding	12	7	5	NS

NS, not significant.

Overall hospital mortality was 50%. There was a trend toward improved ICU outcome for patients in group 2 (*p *= 0.059). Outcome data are shown in Tables 3 and 4. Overall non-survivors spent significantly more time on intermittent positive-pressure ventilation (17 versus 12 days; *p *= 0.003), but overall length of stay in the ICU was not longer.

**Table 3:** Duration of intermittent positive-pressure ventilation and intensive care unit stay in days: comparison between groups

	Duration of IPPV	Duration of ICU stay	Duration of IPPV in survivors	Duration of ICU stay in survivors
All patients	15 (3–54)	17.5 (8–54)	12 (3–39)	18 (8–48)
Refractory coagulopathy (*n *= 25, *n *= 11^a^)	16 (6–54)	19 (13–54)	13 (6–39)	19 (14–48)
Mild or no coagulopathy (*n *= 35, *n *= 24^a^)	13 (3–54)	17 (8–54)	11.75 (3–32)	19 (14–48)

Data are presented as median (and range). ^a^ICU survivors. ICU, intensive care unit; IPPV, intermittent positive-pressure ventilation.

**Table 4:** Intensive care unit and hospital survival

	ICU survival	Hospital survival
All patients	35 (58%)	30 (50%)
Refractory coagulopathy (*n *= 25, *n *= 11^a^)	11 (44%)	10 (40%)
Mild or no coagulopathy (*n *= 35, *n *= 24^a^)	24 (69%)^b^	20 (57%)

Data are presented as median (and percentage). ^a^ICU survivors. ^b^*p *= 0.059. ICU (intensive care unit).

**Figure 1:**
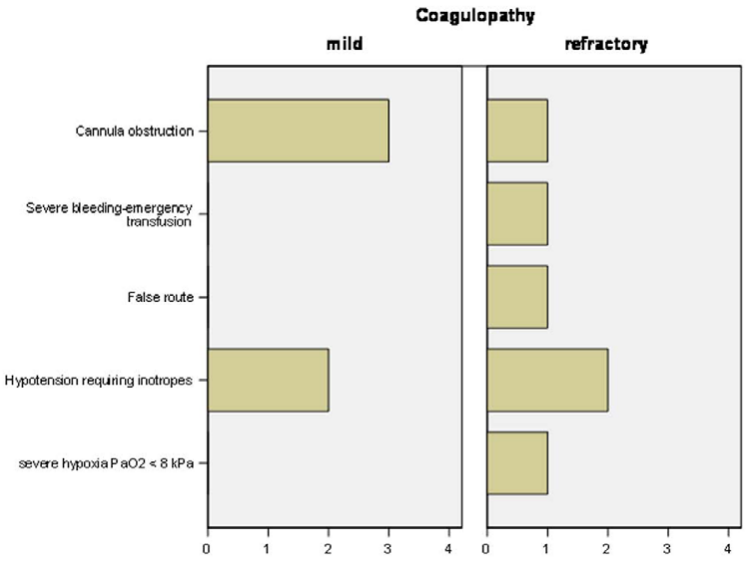
Incidence of severe or life-threatening immediate or late complications. Bars denote number of patients (x-axis). PaO_2_, arterial partial pressure of oxygen.

**Figure 2:**
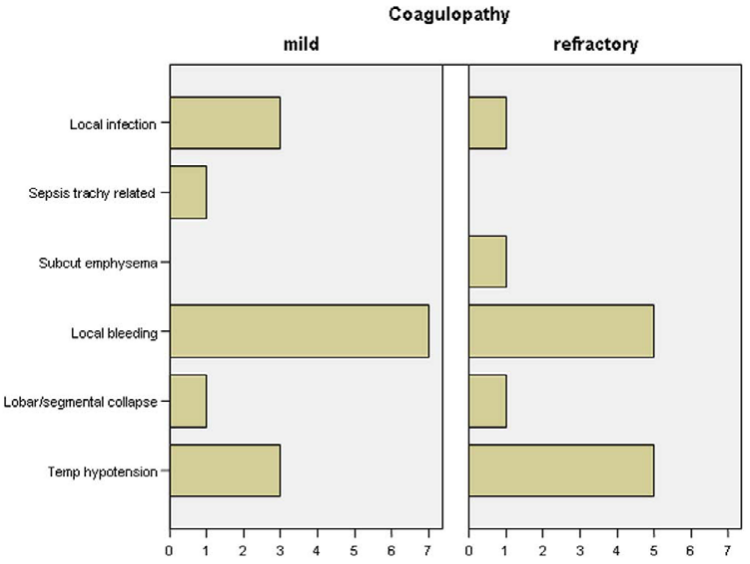
Incidence of minor immediate or late complications. Bars denote number of patients (x-axis).

There was no procedure-related death or peri-procedural cardiac arrest. One patient in the refractory coagulopathy group required emergency transfusional and clotting support with 1 unit of red pack cells, 3 units of FFP, 1 unit of pooled platelets, and cryoprecipitate due to significant bleeding from a pre-tracheal vessel, the only early life-threatening complication. Bleeding stopped after cannula insertion, and no surgical intervention was required. This individual also accounted for the only incidence of significant hypoxaemia during the procedure. Two patients in each group previously not on vasopressor medication experienced hypotension not responsive to intravenous fluid administration and required vasopressor support during or shortly after tracheostomy. One patient in the coagulopathy group had a false route cannula insertion that was immediately recognized and dealt with appropriately. There was no case of conversion to an open surgical technique. Three patients in the mild coagulopathy group and one in the refractory coagulopathy group suffered from life-threatening late complications, all cannulae obstructions. They were readily recognized and all patients survived to ICU discharge, and three were discharged from the hospital. Complications are listed in Figures 1 and 2. Cardiovascular indices, including MAP, central venous pressure, cardiac index, and ITBVI, were not different between groups, nor was EVLWI.

### Clotting support

INR and platelet count were significantly different between groups at all time points (Table 5). Haematocrit was lower in group 1 patients 24 hours following tracheostomy (26.5 versus 27.3%; *p *= 0.013) but not at any other time point. Quantity of FFP and number of platelets transfused immediately prior to PDT were higher in group 1, but this did not reach significance; however, the total amount of platelets administered during the observation period was higher in patients with refractory coagulopathy. There was a trend toward increased FFP administration in this group. The numbers of patients requiring FFP (12 versus 10) and platelet support (21 versus 20) were higher in the refractory coagulopathy group, but this was significant only for the number of patients receiving platelet transfusions (Table 6).

**Table 5:** Clotting profile

Variable	All (*n *= 60)	Refractory coagulopathy (*n *= 25)	Mild coagulopathy (*n *= 35)	*P *value
INR 0	1.21 (0.8–3.7)	1.38 (0.9–3.7)	1.2 (0.8–1.6)	0.03
INR 24	1.18 (0.8–2.36)	1.32 (0.9–2.36)	1.13 (0.8–1.6)	0.01
INR 48	1.2 (0.8–2.46)	1.31 (0.96–2.46)	1.11 (0.8–1.6)	0.003
INR 72	1.2 (0.9–2.2)	1.36 (1–2.2)	1.11 (0.9–1.5)	0.002
Platelet 0	49 (3–477)	30 (3–142)	74 (23–477)	<0.001
Platelet 24	67 (4–411)	37 (4–114)	96 (30–411)	<0.001
Platelet 48	71 (2–447)	39 (2–85)	92 (33–447)	<0.001
Platelet 72	65 (3–416)	43 (3–89)	92 (20–416)	<0.001

Clotting profile prior to percutaneous dilational tracheostomy 0 and at 24, 48, and 72 hours following the procedure. Values are expressed as median (and range). INR, international normalized ratio.

**Table 6:** Clotting support

Variable	All (*n *= 60)	Refractory coagulopathy (*n *= 25)	Mild coagulopathy (*n *= 35)	*P *value
FFP prior to PDT	66	39 (59%)	27 (41%)	NS
Platelets prior to PDT	77	40 (52%)	37 (48%)	NS
FFP total	125	78 (62%)	47 (38%)	0.059
Platelets total	129	80 (62%)	49 (38%)	0.009
Random platelet concentrates	121	58 (48%)	63 (52%)	NS
Patients requiring platelets	41	21	20	0.029
Patients requiring FFP	22	12	10	NS

Units of blood, platelet transfusions, and FFP administered prior to PDT and in total and number of patients requiring clotting products. Number in parenthesis denotes percentage of all units transfused. FFP, fresh frozen plasma; NS, not significant; PDT, percutaneous dilational tracheostomy.

## Discussion

In this prospective study, we showed a low rate of clinically significant procedure-related bleeding complications in patients with liver disease and following liver transplantation, despite a high incidence of refractory coagulopathy. Only one patient in the coagulopathy group who suffered from disseminated intravascular coagulation at the time the tracheostomy was performed bled significantly (mainly extratracheally and greater than 150 mL) and required emergency transfusional and clotting support; no open surgical revision was necessary.

Previous studies commenting on the safety of percutaneous tracheostomy in patients with coagulopathy either were retrospective in nature [7] or looked at patients with a wide range of potential contraindications for PDT, including clotting abnormalities [3,4,8,9]. In other prospective studies or randomized trials, uncorrectable coagulopathy was a relative contraindication for study inclusion [10-12].

Kluge and colleagues [7] retrospectively analysed their experience of the safety of PDT in medical patients with severe thrombocytopenia over the course of a 6-year observation period. Forty-two patients were found to be severely thrombocytopenic (mean platelet count, 26 × 10^9 ^cells/L). Only two patients suffered from significant post-procedural bleeding requiring surgical intervention.

Beiderlinden and colleagues [8] showed a low incidence of relevant bleeding in a prospective trial of 136 PDTs in which 18 patients had significant coagulopathy. In a subsequent study, the same authors reported on the outcome of 203 consecutive PDTs, including 55 patients with a platelet count of less than 60 × 10^9 ^cells/L and a bleeding rate of 6% [9].

Recently, Ben Nun and colleagues [3] reported their experience with PDT in 157 consecutive patients, more than a third of whom (*n *= 55) had conditions referred to as absolute or relative contraindications to the procedure in previous series. Twelve patients had significant coagulopathy that did not normalise despite clotting factor administration or discontinuation of anticoagulation therapy. Despite bleeding complications occurring more frequently in the coagulopathy/anticoagulation group, these were all clinically not relevant.

The difference of our study compared with previous investigations lies in the stringent *a priori *definition of refractory coagulopathy and the sole inclusion of patients with liver-related disease processes. Only patients with a repeat INR of greater than or equal to 1.5 and/or a platelet count of less than or equal to 50 × 10^9 ^cells/L despite clotting support on the day of and the 3 days following the procedure were classified as refractory coagulopathic. Despite the rigorous definition criteria, 42% of patients in our study suffered from non-correctable clotting abnormalities. Including patients with a platelet count of less than 50 × 10^9 ^cells/L or an INR of greater than 1.5 on the day the tracheostomy was carried out, the incidence of severe coagulopathy would have risen to 67% (40 patients). An additional two patients who were only mildly coagulopathic on the day of the procedure became severely coagulopathic during the 72-hour observation period but were analysed within group 2.

The overall complication rate may appear high compared with previous investigations. However, the incidence of clinically important side effects was low. We also tried, *a priori*, to define complications that are clinically less relevant and might have been overzealous by including minor side effects not reported in other studies, such as temporary intraprocedural arterial hypotension. One patient accounted for three (severe bleeding, hypotension requiring vasopressor support, and severe hypoxaemia) of a total of seven severe early complications and the only immediate life-threatening incident (massive extratracheal bleeding) during the trial period.

At the time the study was carried out, we did not use routine bronchoscopic guidance for PDT. Hence, we are unable to comment on the possible increased risk of intratracheal bleeding in an at-risk patient population. However, any significant intraluminal haemorrhage should have been evident on routine post-procedural suctioning or should have caused a significant incidence of segmental or lobar lung collapse visible on chest radiography, neither of which we were able to show. It is unlikely that a posterior tracheal wall laceration remained undiagnosed given the lack of clinically obvious barotrauma complications in the study patients, apart from one incidence of temporary subcutaneous emphysema.

The overall ICU and hospital mortality in this study might appear high given the APACHE II (Acute Physiology and Chronic Health Evaluation II) scores (median of 19 for the whole group). However, SOFA scoring has been shown to be a better predictor of outcome in patients with decompensated liver disease requiring organ support [13]. In the study by Wehler and colleagues [13], a SOFA score of greater than or equal to 9 was associated with an 88% in-hospital mortality in patients with cirrhosis admitted to a medical ICU. The average SOFA score of the patients in this study was 13, and a third of the patients had chronic liver disease as their underlying pathology.

## Conclusion

We conclude that refractory coagulopathy associated with liver disease is not a contraindication for PDT. To the contrary, the relative atraumatic nature of the procedure compared with the open surgical approach makes it an attractive alternative in this setting. Provided adequate clotting support is given and in the hands of experienced operators, PDT can be safely performed in patients with advanced liver disease and refractory clotting abnormalities.

## Key messages

• Percutaneous dilational tracheostomy in patients with liver disease or following liver transplant for acute liver failure suffering from coagulopathy is safe.

• Clinically relevant bleeding complications were infrequent, even in patients with refractory coagulopathy.

• No significant difference in adverse events was found comparing patients with refractory and mild coagulopathy.

## Abbreviations

EVLWI = extravascular lung water index; FFP = fresh frozen plasma; ICU = intensive care unit; INR = international normalized ratio; ITBVI = intrathoracic blood volume index; MAP = mean arterial pressure; PDT = percutaneous dilational tracheostomy; SOFA = Sepsis-related Organ Failure Assessment.

## Competing interests

The authors declare that they have no competing interests.

## Authors' contributions

GA, JWA and GPO made contributions to conception, design, and acquisition of data and were involved with the clinical aspects of the study. GA and WB were concerned with analysis and interpretation of data. All authors were involved with the clinical aspects of the study. All authors were involved in drafting and revising the final manuscript.
